# Unfairness of Random Access with Collision Avoidance in Industrial Internet of Things Networks

**DOI:** 10.3390/s21217135

**Published:** 2021-10-27

**Authors:** Marek Miśkowicz

**Affiliations:** Department of Measurement and Electronics, AGH University of Science and Technology, 30-059 Kraków, Poland; miskow@agh.edu.pl; Tel.: +48-12-617-50-49

**Keywords:** industrial internet of things, access protocols, carrier sense multiaccess, memoryless backoff, modeling, communication system performance

## Abstract

This paper is focused on the analysis of unfairness of random media access in Local Operating Networks (LON), which is one of the commercial platforms of the Industrial Internet of Things (IIoT). The unfairness in accessing the LON channel is introduced by a collision avoidance mechanism in the predictive *p*-persistent CSMA protocol adopted at the media access control layer. The study on the bandwidth share in predictive *p*-persistent CSMA calls for the analysis of multiple memoryless backoff. In this paper, it is shown that the channel access in LON systems is unfair in the short term for medium traffic load conditions, and in the long term for heavy loaded networks. Furthermore, it is explained that the average bandwidth allocated to a particular node is determined implicitly by the load scenario, while an actual node bandwidth fluctuates in time according to stochastic dynamics of the predictive *p*-persistent CSMA. Next, it is formally proven that the average bandwidth available to a node is a linear function of its backoff state and does not depend on backoff states of the other stations. Finally, it is demonstrated that possibly unfair bandwidth share in LON networks determined implicitly by load scenario is stable because, with lowering a fraction of actual network bandwidth accessible by a given station, the probability to decrease it in the future also drops.

## 1. Introduction

Fairness is a concept generally attributed to allocation of limited resources among a set of agents or individuals [1]. The importance of fairness issues in resource allocation has been recognized and well studied in a variety of research problems [1,2,3,4], along with applications in network engineering [5,6,7,8,9]. For communication services, a problem of fair sharing concerns all scarce resources, including link and flow scheduling, channel assignment, rate allocation, congestion control and routing protocols [5]. In an egalitarian vision, fairness simply means allocating the same share to all users, while any scheme resulting in uneven allocation of resources is recognized unfair.

Guaranteeing fairness in accessing the communication channel is a significant issue in distributed computing systems aimed to allocate network resources equitably. Sharing the network bandwidth in random access networks is fair only if the contending nodes have equal chances to possess the channel. Nevertheless, in many random access protocols the contention is, or in some scenarios could be, unfair. Inequitable sharing network resources causes a discrimination of some users implying a decrease of a fraction of bandwidth available to them, and a corresponding increase of their access delays. Due to usual demand on real-time operation, possible unfairness in network access is a more severe issue in networked control systems and Industrial Internet of Things than in conventional data computer networks. In general, a guarantee of fair access concerns the network with medium or heavy traffic load since inequality in sharing the bandwidth is not a problem when the channel is mostly idle, simply because the bandwidth is then not a scarce resource [6,7].

### 1.1. Unfairness of Network Access

A fair network access has to be guaranteed on various levels of the network protocol stack. Unequal chances to win the contention among the nodes might be introduced by the Physical Layer since different distances of devices from the transmitting station in the spatially distributed network yield distinct propagation delays of a signal in a communication medium. As a result, at the end of the transmission, some nodes sense the channel to be idle, and the others may detect the channel still to be busy. To resolve this problem, in the slotted random access protocols, the contention slot width and the minimum interpacket time are adjusted to the maximum end-to-end propagation delay [10].

Unfair access may appear in underwater acoustic sensor networks (UASN) due to slow propagation of sound speed in water (1500 m/s). The requests sent by the nodes that are far from the receiver may be processed later than the requests transmitted by the nodes that are close to the receiving node. This implies unfair channel access and calls for development of specialized protocols for UASN resolving spatial-temporal uncertainty [11].

A fair sharing network bandwidth belongs essentially to the tasks of Media Access Control (MAC) protocols designed to allocate network resources among the nodes equitably. Some communication standards in Industrial Internet of Things use *priority systems* that introduce the *intended* inequality in dividing the limited network resources among the stations to favor transmission of messages produced by distinct nodes, or of urgent messages in general.

Unfairness might be an unavoidable effect of the media access control mechanism used. An example is the *dominance protocol* adopted to resolve the collisions in Carrier Sense Multiple Access (CSMA) schemes (e.g., in Controller Area Networks (CAN) or Inter-Integrated Circuit (I^2^C) serial communication bus). This protocol has a good total throughput (no waste of bandwidth due to collisions), high efficiency and offers global prioritization at the cost of unfairness in sharing communication resources. In particular, if the usage of shared channel is not limited, the node with high priority can monopolize the network resources.

Furthermore, unfairness may appear in heterogeneous sensor networks (HSNs) if the applications generate data packets of a different size (e.g., temperature, humidity, or images) implying unequal bandwidth share [8,12]. Fairness can be also strongly disturbed under network attacks [12,13]. For example, under multiple access attacks (e.g., backoff attack) misbehaving stations would achieve a larger portion of the network bandwidth at the expense of the stations that respect the regular MAC mechanism [13].

Conversely, unfairness may result from mechanisms aimed to maximize the network throughput. In particular, in random access protocols, unfair sharing channel bandwidth is often introduced by collision avoidance schemes based on feedback from the network. A representative example is the IEEE 802.11 protocol for WLANs. Resetting the contention window size *CW* to its minimum value *CW*_min_ after each successful transmission leads to a short-term unfairness since a winning station will be privileged to win the contention on its next attempt. If the network is heavily loaded, the other stations will suffer severe throughput degradation resulting in unfair share of wireless channel bandwidth. Thus, binary exponential backoff in the IEEE 802.11 favors the winning stations to continue winning the contention in the near future [6,7].

Fairness and system efficiency are usually contradictory objectives [1,2]. A scheme that maximizes throughput without regard to treating individual flows equitably is regarded as fully “efficient”. If fairness considerations are incorporated, the efficiency of the system is likely to decrease. The tradeoff between fairness and system efficiency is a common issue and concerns not only communication systems [2,9,12,14]. The efficiency loss (i.e., the difference between the maximum system efficiency and the efficiency under the fair scheme) relative to the maximum system efficiency can represent the “price of fairness” [3]. Some approaches are aimed to maximize both the system efficiency (e.g., throughput) and fairness [14]. A variety of mechanisms to increase fairness in Internet-of-Things-based networks has been proposed and examined in recently published works [12,13,15,16].

### 1.2. Paper Contribution

The present study addresses a problem of the unfairness in accessing the channel introduced by a random access scheme adopted in Local Operating Networks (LON), one of communication platforms that belong to Industrial Internet of Things (IIoT). LON has become a classical solution in building automation, home networking, street lighting control, and smart cities [10].

The media access control mechanism adopted in LON in its non-priority phase, called the *predictive p*-*persistent CSMA*, belongs to the class of MAC protocols aimed essentially to share average bandwidth equitably to all the nodes in the network. The predictive *p*-persistent CSMA scheme uses the collision avoidance mechanism similar to the IEEE 802.11 protocol. The collision avoidance allows to keep high total network throughput even under overload conditions by scaling the backoff to the expected traffic load [17]. However, as it will be shown in this paper, the collision avoidance causes sharing network bandwidth among the stations inequitably as a side effect of maximizing the total throughput. The predictive *p*-persistent CSMA is thus the example of a MAC protocol that sacrifices fairness for better efficiency beside the IEEE 802.11 [6,7], or Dynamic Channel Negotiation MAC (DCN-MAC) for Underwater Acoustic Sensor Networks [11].

Unlike IEEE 802.11 protocol, unfairness of the predictive *p*-persistent CSMA except preliminary study in [18], has not been yet indicated and analyzed neither in the technical documentation, nor in engineering literature. Meanwhile, the problem of unequal access is significant since, as follows from the forthcoming discussion, the unfairness is an inherent part of the predictive *p*-persistent CSMA operation in most network traffic scenarios. Thus, the present work contributes to rapidly growing research on fairness of various network services including Internet-of-Things-based networks.

The study on the predictive *p*-persistent CSMA protocol fairness calls for the analysis of *multiple memoryless backoff*. In the literature, there are many works that deal with the multiple non-memoryless backoff since it models the operation of Distributed Coordination Function (DCF) mechanism in the IEEE 802.11 (e.g., [19]), and for example also in Sensor MAC (SMAC) [20], WiseMAC [21], Timeout MAC (TMAC) [22], and Dynamic Sensor MAC (DSMAC+) [23]. Lower attention has been devoted to the analysis of the multiple memoryless backoff [17,18,24,25,26,27,28]. The latter has been adopted not only in the predictive *p*-persistent CSMA but also in the IEEE 802.3 (Ethernet) and IEEE 802.15.4 protocols [29,30]. In order to investigate the access unfairness, this paper is focused on the examination of stochastic dynamics of the predictive *p*-persistent CSMA. The presented research addresses four main aspects:

- specification of reasons that evoke the unfairness of the network access in the predictive *p*-persistent CSMA;
- analysis of possible unequal bandwidth distribution among the contending nodes;
- impact of unequal bandwidth distribution on the total network utilization that represent system efficiency;
- examination of the bandwidth allocation stability in time aimed to assess if unfairness tends to escalate or is under self-imposed limit.

The original contributions made by this paper to the state of the art are as follows. First, it is shown that the channel access in the predictive *p*-persistent CSMA is unfair in short term for medium traffic load conditions, and in long term for heavy loaded network. Second, it is explained that the average bandwidth allocated to a particular node is determined implicitly by the load scenario (i.e., rate and specification of input traffic generated to the network by a particular node and other contending nodes), while an actual node bandwidth fluctuates in time according to stochastic dynamics of the predictive *p*-persistent CSMA. Next, it is formally proven that the average bandwidth available to a node is a linear function of its backoff state and does not depend on backoff states of the other stations. Finally, it is demonstrated that possibly unfair network bandwidth share in LON networks determined implicitly by load scenario is stable in time because, with lowering a fraction of actual network bandwidth accessible by a given station, the probability to decrease this actual bandwidth in the future also drops. The presented analysis is based on qualitative measures of fairness.

## 2. Fairness in LON Networks

Local Operating Networks (LON) platform is one of leading standards in Industrial Internet of Things addressed to a wide range of applications. Thus far, tens of millions of devices have been installed with LON technology worldwide. In particular, LON has become a classical solution in building automation, home networking, street lighting and smart cities [10].

### 2.1. Reason of Unfairness

LON networked control systems are built upon event-driven architecture [10] and adopt event-triggered transmission strategy [31,32]. One of efficient services employed in LON platform to improve utilization of the network bandwidth is *multicast addressing*. This service is rare in industrial networking. A multicast message is received by a group of destination nodes. Thus, multicast transactions save communication bandwidth since a single multicast (one-to-many) message can substitute a set of unicast (one-to-one) messages addressed to each recipient individually [33].

Because the random access protocol of CSMA type used in the LonTalk MAC sublayer does not provide guarantee of message delivery, the classical acknowledged message service is used by default to improve end-to-end communication reliability. As a response to the acknowledged multicast message reception, each receiver generates a positive acknowledgement to the sender. In the LonTalk random access protocol, the acknowledgement packets contend for the channel access together with messages since there is no dedicated collision-free channel for sending acknowledgements as it is in the IEEE 802.11. Thus, soon after a successful transmission of a multicast acknowledged message, a number of nodes equal to the number of multicast message recipients almost at the same time try to access the channel to send the acknowledgement packets additionally to the traffic of the original messages or stale acknowledgements. To avoid excessive collisions due to a burst of acknowledgements, a collision avoidance with a prediction mechanism is used in the LonTalk random access protocol. The prediction is based on accumulating the numbers of expected acknowledgements (or responses) encoded in the header of each message sent or received, and on accumulating the number of expected message retransmissions by detecting collisions if hardware collision detection is provided. Each node predicts the expected traffic autonomously using *backlog counters* implemented in the protocol. The actual number of contention slots used by each node to randomize the network access is proportional to its current backlog state.

The fundamental problem is that there is no global coordination of states of backlog counters among the nodes. Since the backlog increments encoded in the packet headers and used for traffic prediction is distributed through the network only by successfully transmitted messages, the states of backlog counters are consistent any time provided that the channel is noise-free and collision-free, or at least if unsuccessful transmissions are recognized by all the nodes (not only transmitters of messages involved in collision). Otherwise, the numbers of contention slots used to randomize the channel access may vary among the contending nodes, which favors the node(s) with the lowest backlog state to win the contention. The contending nodes with higher backlog states have lower chance to possess the channel. Thus, the inconsistency of backlog states results in unfairness of channel access in LON networks.

### 2.2. Protocol Specification

The random access scheme in the LonTalk protocol, registered as the ANSI/CEA-709.1-B standard, is the predictive *p*-persistent CSMA that belongs to variable-window slotted-CSMA protocols [17,33]. A node contending for a channel selects a random backoff expressed as a random number of slots drawn from the uniform distribution between 1 and *W*, where *W* is the size of the contention window. If the channel is still idle when the random delay expires, the node transmits in slot *r*. Otherwise, the node receives an incoming packet, discards the slot drawn and competes for the channel access again. If more than one node chooses the same slot number, and when that slot has the lowest number selected by any node with a packet to send, then a collision happens. All the packets involved in a collision are corrupted.

In the predictive *p*-persistent CSMA scheme, the size of the contention window managed by each node autonomously, varies in time and is dynamically adjusted to the expected channel load. The number of slots grows by factor *BL*, called the *estimated backlog* since *W* = 16*BL*, where *BL* is the actual state of the backlog counter that cannot be lower than one, and higher than 63.

The predictive *p*-persistent CSMA scheme belongs to variable-window CSMA protocols with *memoryless backoff*, which means that the contenders draw a number of a contention slot in every transmission attempt anew and cancel them when the transmission is detected in the channel). For a comparison, in the IEEE 802.11, the non-memoryless backoff is applied where the competing nodes freeze their backoff timers in case of detecting transmission, wait to the end of the current packet transmission, and then resume these timers.

On the top of the predictive *p*-persistent CSMA, the acknowledged and the unacknowledged message services are provided. As a response to an acknowledged message reception, each receiver generates a positive acknowledgement packet to the sender. The message may be addressed to a single recipient (unicast), or to a group of recipients (multicast). In the unacknowledged service, the acknowledgements are not applicable. In the LonTalk, the acknowledgement packets compete for the channel together with messages according to the same contention algorithm.

### 2.3. Backlog Counting Algorithm

As stated, the backlog estimation is based on predicting the current number of packets expected in a channel contention. The actual state of the backlog counter *BL* varies from one to the next transmission attempt and relies on the accumulation of consecutive backlog increments and decrements [17,33].

**Figure 1:**
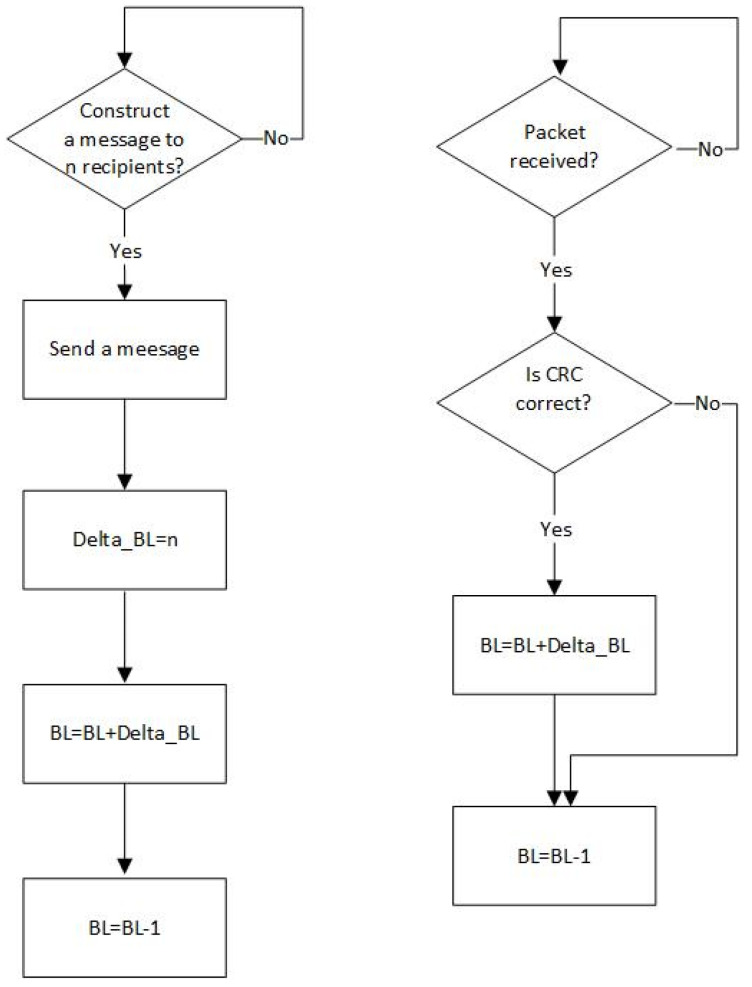
Backlog counting algorithm for transmitting nodes (**left** diagram), and for receiving nodes (**right** diagram) if the nodes are not equipped with collision detection.

Backlog counting, built into the node firmware, consists in the following principles: (i) successive backlog increments based on the information included in the header of each packet sent or successfully received by a particular node; this information is encoded in the 6-bit long data field *Delta_BL*; (ii) successive backlog decrements by one at the end of packet transmission provided that the collision is not detected (Figure 1).

Thus, the backlog *BL* is increased after successful packet transmission or reception by a *Delta_BL*-1 where *Delta_BL* represents the number of acknowledgements that will be generated by receiver(s) as a result of a successful packet reception. For unicast messages *Delta_BL* equals one, and for multicast messages *Delta_BL* is higher than one but lower than 63 which is the maximum size of a group of receiving nodes addressed by a single multicast message. Consequently, the *Delta_BL* equals zero either for the unacknowledged messages, or for the acknowledgement packets. The data field *Delta_BL* is read by each node in the network, where a packet is broadcasted before examination of packet destination address(es).

### 2.4. Aim of Collision Detection

**Figure 2:**
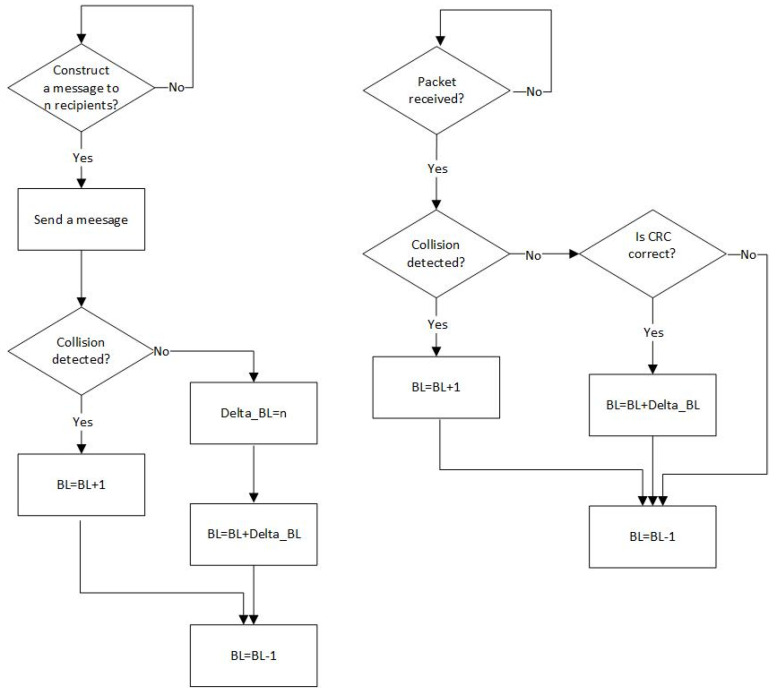
Backlog counting algorithm for transmitting nodes (**left** diagram) and receiving nodes (**right** diagram) if they are able to detection collisions.

Optionally, the backlog counter might be also incremented by one in case of a collision if the nodes are equipped with the collision detection hardware [17] (Figure 2).

In traditional computer networks, the collision detection has been provided to avoid a waste of bandwidth by stopping as soon as possible transmissions of long data packets involved in collisions [34]. This argument is invalid in networking technologies that form Internet of Things since the size of packets transmitted through the channel is relatively small and usually does not exceed a few dozens of bytes. The motivation to use the collision detection in LON networks is different from that related to computer data networks. First, from the point of view of the packet sender, the collision detection reduces the response time because the sender does not have to wait for time-out before attempting to resend the messages. Second, in the context of global network performance, the collision detection is a part of collision avoidance mechanism that improves the network throughput due to adjustments of the contention window size to the current traffic load [17].

## 3. Providing Fairness in Random Access Protocols

Fairness in distributed computer systems has been studied since origins of network engineering using both qualitative and quantitative metrics. The first widely accepted quantitative measure of fairness was proposed by Jain [35]. The Jain’s fairness index evaluates the equality of user allocation and is bounded between 0 and 1. A totally fair resource allocation system has a value of Jain’s fairness index equal to 1, while a totally unfair system reaches a value of 0. Jain’s index is intended to evaluate long-term fairness but can be also used for classifying short-term fairness by applying sliding window averages of resource share [7]. Although Jain’s index reflects well the fairness of the whole system, it does not provide a deeper view to relationships between fraction of resources allocated to particular users [5]. The other measure that has been adopted for quantitative evaluation of fairness is entropy introduced in the context of information theory [36]. Entropy may be considered as expression of fairness because it is maximized when the allocation is equal [37].

Qualitative measures do not evaluate fairness by numbers but instead they give guidance for possible fair resource allocation. Two most representative qualitative measures are max-min and proportional fairness. A resource allocation is *max–min fair* if an attempt to increase the allocation of any flow must be at the cost of a decrease of some already smaller flow. The max-min fairness is used in various areas of networking and aims at allocating as much as possible resources to users with low rates and, at the same time, not unnecessarily wasting available resources [4]. Conversely, the proportional fairness is the ratio between the maximum and the average resource consumption [37].

The aforementioned approaches to fairness assessment that may be classified as based on Quality-of-Service (QoS) of the network are insufficient to examine fairness related to Quality-of-Experience (QoE) of end users. Although QoE of a network service user is in general dependent on QoS of the network, the notions of fairness commonly applied in the QoS domain do not translate well to the QoE domain [38]. In [39], the QoE fairness metric is defined as a linear transformation of the standard deviation of QoE of all users consuming a service to the range of [0; 1], and the system is absolutely QoE fair when all users receive the same QoE value.

Considering the time duration, fairness can be categorized into short term and long term [6,7]. A system is said to be *long-term fair* if all the users gain equitable access to its resources in the long-run, although there may be transient periods of unbalanced access. *Short-term fairness*, instead, refers to equitable share of resources in short run. Usually, short-term fairness implies long-term fairness but not vice versa [6,7].

### 3.1. Fairness of Random Access

To discuss a possible inequality in distributing shared resources in the random access protocols, we choose the probability of successful transmission of a given contending node as a bandwidth allocation metric. If this probability is the same for all the contending nodes in each transmission attempt, then the network access is fair. It can be also easily demonstrated that equal probability of successful transmission for each node implies even throughputs and the mean access delays among contending stations [17,27].

It is evident that the channel access according to slotted CSMA schemes with memoryless backoff is fair if all contenders choose a given contention slot used to settle the competition with equal probability. According to this observation, the probability of choosing slots within the contention window might vary among the contention slots (e.g., in the CSMA scheme with a geometrical distribution [40,41]) but particular slots have to be drawn by the contenders with the same probability. From the above condition, the following straightforward conclusion can be derived for the predictive *p*-persistent CSMA.

**Remark** **1.** *Channel access according to the predictive p-persistent CSMA protocol is fair if and only if all the contending nodes have the same backlog states before each transmission attempt*.

### 3.2. Providing Backlog Consistency in Predictive p-Persistent CSMA

As stated, there is no global coordination of states of backlog counters among the nodes. Each node computes the channel backlog autonomously based on its backlog counter. To keep the consistency of backlog states as required in Remark 1, all the nodes in the network should modify their backlog counters by the same increment or decrement synchronously before each transmission attempt.

The backlog counters consistency is guaranteed if the channel is noise-free, all the nodes are equipped with collision detection and collision detection technique allows every node to recognize each collision occurrence in a channel [17]. The backlog state is then the same for all the nodes in the network and varies randomly in time due to stochastic nature of the predictive *p*-persistent CSMA scheme. In the network steady state, the probability density function of backlog state is unimodal for the protocol version with collision detection, and quasi-exponential for the version without collision detection [17]. Both in the protocol version without and with collision detection, the backlog probability distribution is long-tailed. The analysis of the predictive *p*-CSMA performance based on queuing theory is reported in [24,25].

However, in the presence of transmission errors due to noise, or if the collision detection is absent, or if the collision detection is provided but the nodes are unable to recognize all collision occurrences in a channel, the backlog states can differ among the nodes as follows from a description of the backlog counting algorithm stated in Section 2.1.

First, the packets with an invalid CRC introduce the backlog inconsistency since all the nodes in a network segment where a packet is broadcasted reject it, and only the packet sender modifies the backlog counter according to the content of its *Delta_BL* field. Thus, the noise introduced by Physical Layer can break backlog consistency since recipients cannot read *Delta_BL* field of corrupted packets. For example, if the multicast message to 3 recipients is corrupted by the noise, the sender finally increments its counter by 2, and all the other nodes decrement their backlog counters by 1.

Second, the backlog inconsistency is introduced by Link Layer, if the nodes are unable to recognize unsuccessful transmissions (i.e., collision detection is not provided, or the nodes are unable to detect all collisions). Namely, if a packet is involved in a collision during transmission in the channel without collision detection, the modifications of backlog counters, introduced by the senders and by the remaining nodes in the network, finally differ. The senders after transmission increase their counters by the *Delta_BL*. However, all the other nodes in the network are unable to read the number encoded in the *Delta_BL* data field of the packets corrupted by collisions; thus, their backlog counters are decremented by one according to the backlog counting principles presented in Section 2.1. For example, if the multicast message addressed to two recipients collide with the unicast message, then their senders increment the corresponding counters by 1 and zero respectively, whereas all the other nodes decrement their backlog counters by 1. The senders of collided packets have a lower chance to win the contention in future since their backlog states will be larger than the backlog of the other nodes. The unfairness introduced by the collision is greater, if the multicast messages collide. In particular, the collision does not cause the unfairness if the packets involved in a collision have *Delta_BL* equal to zero (e.g., if the unacknowledged messages collide with the acknowledgment packets). Moreover, if the input traffic does not contain acknowledged multicast messages in the channel without collision detection, then the Link Layer does not introduce backlog inconsistency since the predictive *p*-persistent CSMA scheme is then reduced to the pure 0.0625-persistent CSMA where the traffic-based contention window adaptation is disabled [27]. However, the price of fair channel access in this load scenario is the absence of the collision avoidance mechanism.

The backlog inconsistency is introduced also if the collision detection is provided, but only senders of packets involved in collision are able to detect it. The senders increment their backlog counters by one but all the other nodes in the network segment decrease their backlog counters by one. Thus, such a *partial* collision detection also introduces backlog inconsistency. Unfortunately, the LON transceivers that were available on the market enable only senders to detect possible packet collisions (like Ethernet transceivers) and increment their backlog counters. Meanwhile, the role of collision detection in LON control networks is more extensive than in conventional LANs as stated in Section 2.3.

### 3.3. Short-Term and Long-Term Unfairness

The inconsistency in backlog counting is not a serious problem if a channel stays under light or medium traffic load, because idle cycles can recover the backlog consistency if the minimum window size (*BL* = 1) is reached for some time. More specifically, the fairness is recovered not later than after a number of idle transmission cycles equal to the highest backlog state possessed by any node. Thus, if the network is not heavily loaded, the unfairness in LON systems is short term. However, if the channel is heavy loaded, there are no idle cycles and the minimum backlog state equal to one cannot be reached. Since there is no mechanism that forces coordination of backlog counters among the nodes, the unfairness in channel access is introduced in the long run although accidentally the consistency of states may occur.

### 3.4. Implicit Bandwidth Allocation in Predictive p-Persistent CSMA

In a result of protocol unfairness, the bandwidth share in LON networks is dynamical and determined by the random evolution of the backlog counters in contending nodes. The relations between fractions of bandwidth available to particular nodes change in time. A node that transmits many messages is more often exposed to collisions and to decrease its chance to access the channel in future. Moreover, if it transmits many multicast messages, a reduction of allocated bandwidth may be higher. Conversely, the node that is silent, or produces unacknowledged or unicast acknowledged messages potentially has a higher chance to transmit successfully.

The process of bandwidth allocation cannot be controlled by a user but is determined implicitly by the load scenario (i.e., a specification of input traffic generated to the network by particular nodes). A fraction of available bandwidth changes in time according to stochastic dynamics of the predictive *p*-CSMA protocol. In Section 4, a more extensive analysis of the unfairness of the predictive *p*-persistent CSMA is provided.

## 4. Analysis of Backlog Inconsistency Model in Predictive *p*-Persistent CSMA

Since the problem of the protocol unfairness is related especially to the heavy network load, we choose the *saturation network* status as the conditions for investigation of inequality in accessing the channel. The saturation status represents the largest possible load offered by a given number of nodes.

Let us consider a LON network that contains a number of *n* nodes. As described, the state of backlog counters residing at particular nodes evolve stochastically with the estimated channel load. A discrete dynamical stochastic system of distributed backlog counters might be in the steady state, or in the transient state.

As already mentioned, the forthcoming model of backlog inconsistency involves analysis of multiple memoryless backoff [18,26]. The parallel analysis of inconsistency of non-memoryless backoff modeling the network attack by deviation of MAC scheme (backoff attack) in the IEEE 802.11 has been reported in [15].

### 4.1. Basic Backlog Inconsistency Model

At the beginning of the analysis, we propose to examine a basic backlog inconsistency model where the *n* nodes are distributed in two groups. One group contains *n*-*m* nodes occupying the backlog state *k*_1_, the other group includes *m* nodes that are in the state *k*_2_ > *k*_1_. Let us assume arbitrarily that at the beginning of the analysis, all the nodes are in the state *k*_1_ (*m* = 0), and then the backlog states of the nodes successively move from the state *k*_1_ to the state *k*_2_. Finally, in the proposed backlog inconsistency model, all the *n* nodes reach the state *k*_2_ that means in particular a recovery of backlog consistency. Since the proposed model is arbitrary, the analysis does not deal with causes that force such a backlog evolution. Thus, the analysis is valid for any protocol version (either with or without of collision detection). A transition of the backlog state from *k*_1_ to *k*_2_ may occur immediately in one transmission attempt or in a few attempts. In particular, the transition from *k*_1_ to *k*_2_ occurs in a single transmission attempt if the acknowledgement packet(s) or the unacknowledged message(s) collide with the acknowledged message addressed to (*k*_2_ − *k*_1_ + 1) recipients in a channel without collision detection.

The aim of the analysis is to examine: (1) how the distribution of bandwidth available to particular nodes changes with evolution of their backlog states; (2) what is an influence of the backlog state inconsistency on the global network performance represented by the total probability of successful transmission; (3) whether the process of implicit bandwidth allocation in LON systems is stable.

Denote by $p_{succ(1)}^{\left(i\right)}(n-m,m)$ the probability that a certain node occupying the state *k_i_*, *i* = 1; 2 transmits a packet successfully provided that the numbers of nodes residing at the states *k*_1_ and *k*_2_ equal *n* − *m* and *m*, respectively. Both probabilities $p_{succ(1)}^{\left(1\right)}(n-m,m)$ and $p_{succ(1)}^{\left(2\right)}(n-m,m)$ are given by the following formulae [18]:

(1) $$p_{succ(1)}^{\left(1\right)}(n-m,m)=\frac{1}{16k_{1}}\sum_{s=1}^{16k_{1}}{\left(\frac{16k_{1}-s}{16k_{1}}\right)}^{n-m-1}{\left(\frac{16k_{2}-s}{16k_{2}}\right)}^{m}$$

(2) $$p_{succ(1)}^{\left(2\right)}(n-m,m)=\frac{1}{16k_{2}}\sum_{s=1}^{16k_{1}}{\left(\frac{16k_{1}-s}{16k_{1}}\right)}^{n-m}{\left(\frac{16k_{2}-s}{16k_{2}}\right)}^{m-1}$$

for 0 ≤ *m* < *n*.

In Formula (1), the probability $p_{succ(1)}^{\left(1\right)}(n-m,m)$ is defined as the sum of products of the following partial probabilities for each one from 1, …, 16*k*_1_ slots, which are available for the node with the backlog state *k*_1_:

- probability that a winner selects a certain slot *s*, *s* = 1, …, 16*k*_1_, which equals 1/(16*k*_1_);
- probability that the other (*n* − *m* − 1) nodes occupying the state *k*_1_ select one of slots *s* + 1, …, 16*k*_1_ equal to [(16*k*_1_ − *s*)/16*k*_1_]*^n^*^−*m*−1^;
- probability that the *m* nodes occupying the state *k*_2_ select one from the slots *s* + 1, …, 16*k*_2_, which equals [(16*k*_2_ − *s*)/16*k*_2_]*^m^*.

In Formula (2), the probability $p_{succ(1)}^{\left(2\right)}(n-m,m)$ is defined similarly, however the probability that a winner selects a certain slot *s*, *s* = 1, …, 16*k*_2_ equals 1/(16*k*_2_) since it draws from the range 1, …, 16*k*_2_.

In particular, if all the *n* nodes occupy the state *k*_2_ (*m* = 0), then [18]:

(3) $$p_{succ(1)}^{\left(2\right)}(0,n)=\frac{1}{16k_{2}}\sum_{s=1}^{16k_{2}}{\left(\frac{16k_{2}-s}{16k_{2}}\right)}^{n-1}$$

**Figure 3:**
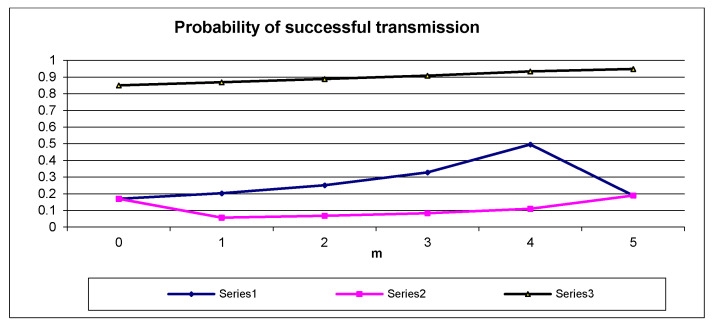
Plots of $p_{succ(1)}^{\left(1\right)}(n-m,m)$, $p_{succ(1)}^{\left(2\right)}(n-m,m)$ vs. number of nodes (*m*) that occupy backlog state *k*_2_ and $p_{succ(1)}^{\left(2\right)}(0,n)$ according to (1), (2), (3), and $p_{succ}(n-m,m)$ according to (9) for *n* = 5, *k*_1_ = 1, and *k*_2_ = 3.

If at least one node occupies the state *k*_1_, then any successful transmission starts in a slot from the range 1, …, 16*k*_1_; thus, the true contention is reduced to 16*k*_1_ slots as expressed by (1), (2). However, if all the nodes are at the state *k*_2_, the contention is carried out within a number of 16*k*_2_ slots; thus, the sum in (3) contains 16*k*_2_ elements. The relationships $p_{succ(1)}^{\left(1\right)}(n-m,m)$, $p_{succ(1)}^{\left(2\right)}(n-m,m)$ versus *m* together with $p_{succ(1)}^{\left(2\right)}(0,n)$ according to (1), (2), (3) for *n* = 5, *k*_1_ = 1, and *k*_2_ = 3 are illustrated in Figure 3.

Comparing Formulae (1) and (2), it is evident that for 0 < *m* < *n*:

(4) $$p_{succ(1)}^{\left(2\right)}(n-m,m)\;<\;p_{succ(1)}^{\left(1\right)}(n,0)\;<\;p_{succ(1)}^{\left(1\right)}(n-m,m)$$

Thus, the backlog inconsistency among the nodes causes a decrease of bandwidth available to the node(s) that has higher backlog state(s), which is represented by the left inequality of (4). At the same time, the bandwidth accessed by the nodes that occupy the lower backlog state is increased as defined by the right inequality of (4).

As seen in Figure 3, the probability of successful transmission for each node that moves its state from *k*_1_ to *k*_2_ decreases because $p_{succ(1)}^{\left(1\right)}(n-m+1,m-1)\;>\;p_{succ(1)}^{\left(2\right)}(n-m,m)$, *m* = 1, …, *n*.

The relative change of the probability of successful transmission for a node due to moving its backlog state is expressed by Remark 2 that will be defined for a general backlog inconsistency model.

### 4.2. Bandwidth Share in General Backlog Inconsistency Model

Generalizing the backlog inconsistency model introduced in Section 4.1, let us assume that a number of *n* contending nodes occupy a number of *l* backlog states *k*_1_, *k*_2_, …, *k_l_* where *k_i_* < *k_i_*_+1_ such that *n_i_* nodes reside in the state *k_i_*, *i* = 1, …, *l* and *n* = *n*_1_ + … + *n_l_*. The probability $p_{succ(1)}^{\left(i\right)}(n_{1},n_{2},\ldots ,n_{l})$ that a certain node that is in the state *k_i_* transmits successfully is defined as:

(5) $$p_{succ(1)}^{\left(i\right)}(n_{1},n_{2},\ldots ,n_{l})=\frac{1}{16k_{i}}\sum_{s=1}^{16k_{1}}\left[{\left(\frac{16k_{1}-s}{16k_{1}}\right)}^{n_{1}}\ldots {\left(\frac{16k_{i-1}-s}{16k_{i-1}}\right)}^{n_{i-1}}{\left(\frac{16k_{i}-s}{16k_{i}}\right)}^{n_{i}-1}{\left(\frac{16k_{i+1}-s}{16k_{i+1}}\right)}^{n_{i+1}}\ldots {\left(\frac{16k_{l}-s}{16k_{l}}\right)}^{n_{l}}\right]$$

Formula (5) is an extension of Formulae (1)–(3). The contention is reduced to the number of 16*k*_1_ slots defined by the lowest backlog state *k*_1_. A winner occupying the state *k_i_* selects the winning slot *s* with the probability 1/(16*k_i_*), the other (*n* − 1) nodes from the *i*th subset choose later slots *s*+1, …, 16*k_i_* with probability $[{\left(16k_{i}-s)/16k_{i}\right]}^{n_{i}-1}$, and all the nodes from the *j*th group (*i* ≠ *j*) choose later slots *s* + 1, …, 16*k_j_* with probability $[{\left(16k_{j}-s)/16k_{j}\right]}^{n_{j}}$. Remark 2 defines the bandwidth share among the nodes for the general backlog inconsistency model.

**Remark** **2.** *The node that moves from the backlog state k_i_ to k_j_, while the other nodes still occupy their states, changes its probability of a successful transmission by the factor k_j_/k_i_*:

(6) $$\frac{p_{succ(1)}^{\left(i\right)}(n_{1},\ldots ,n_{i},\ldots ,n_{j},\ldots n_{l})}{p_{succ(1)}^{\left(j\right)}(n_{1},\ldots ,n_{i}-1,\ldots ,n_{j}+1,\ldots n_{l})}=\frac{k_{j}}{k_{i}}$$

In particular, if *k_j_* > *k_i_*, then the success probability for a node moving its state from *k_j_* to *k_i_*, increases. Conversely, if *k_j_* < *k_i_*, this probability decreases.

In order to prove Remark 2, let us notice that:

(7) $$p_{succ(1)}^{\left(i\right)}(n_{1},\ldots ,n_{i},\ldots n_{i},\ldots ,n_{l})=\frac{1}{16k_{i}}\sum_{s=1}^{16k_{1}}{\left(\frac{16k_{1}-s}{16k_{1}}\right)}^{n_{1}}\ldots {\left(\frac{16k_{i}-s}{16k_{i}}\right)}^{n_{i}-1}\ldots {\left(\frac{16k_{j}-s}{16k_{j}}\right)}^{n_{j}}\ldots {\left(\frac{16k_{l}-s}{16k_{l}}\right)}^{n_{l}}$$

(8) $$p_{succ(1)}^{\left(j\right)}(n_{1},\ldots ,n_{i}-1,\ldots n_{j}+1,\ldots ,n_{l})=\frac{1}{16k_{j}}\sum_{s=1}^{16k_{1}}{\left(\frac{16k_{1}-s}{16k_{1}}\right)}^{n_{1}}\ldots {\left(\frac{16k_{i}-s}{16k_{i}}\right)}^{n_{i}-1}\ldots {\left(\frac{16k_{j}-s}{16k_{j}}\right)}^{n_{j}+1-1}\ldots {\left(\frac{16k_{l}-s}{16k_{l}}\right)}^{n_{l}}$$

By dividing (7) by (8), Formula (6) is obtained.

The content of Remark 2 is surprising since the ratio of both probabilities is independent of the number of contending nodes *n* = *n*_1_ + … + *n_l_* and their deployment among the backlog states *k_i_* (*k_i_* = *k*_1_, …, *k_l_*). An additional result of changing the state of a distinct node is an increase (if *k_j_* > *k_i_*) or a decrease (if *k_i_* > *k_j_*) of the success probabilities for all the other nodes similarly as defined by the right inequality of (4) for the basic backlog inconsistency model. Furthermore, note that the success probability of the distinct node may change in future even if this node will stay in the same backlog state but if the other nodes will change their states. Each active node that moves from one to the other state changes its success probability by the factor equal to the ratio of both states as defined by Remark 2. Thus, the current bandwidth allocation is not determined only by the occupied backlog state but also on the deployment of the other contenders among the states.

### 4.3. Fairness Recovery vs. Channel Load

Let us consider the case when a certain node moves from *k*_1_ to *k*_2_ while all other nodes keep the state *k*_1_. As follows from (6), the probability that the distinct node successfully transmits reaches its maximum if the backlog state is at its minimum (*k*_1_ = 1), which corresponds to the light channel load. Thus, the most significant access unfairness is introduced if the background traffic of the network is low, and the contention window consists of 16 slots (*k*_1_ = 1). However, the fairness recovery is then faster, and may occur even after one transmission attempt. If the background backlog state *k*_1_ > 1, which corresponds to the medium or heavy load conditions, then the unfairness is lower, but the time needed to recover the access fairness is longer and may occur at least after *k*_2_ − 1 transmission attempts.

### 4.4. Fairness vs. Mean Channel Utilization

The backlog inconsistency has also impact on the global network performance (that may be considered as the system efficiency according to [2], or [3]) as demonstrated in the forthcoming discussion. To estimate the global network performance, we choose the total probability of successful transmission of any packet. This probability is defined as the sum of success probabilities of all the contending nodes. If a number of *n_i_* nodes reside in the state *k_i_*, *i* = 1, …, *l* where *k_i_* < *k_i_*_+1_, then the total probability $p_{succ}(n_{1},n_{2},\ldots ,n_{l})$ of successful transmission equals:

(9) $$p_{succ}(n_{1},n_{2},\ldots ,n_{l})=n_{1}p_{succ(1)}^{\left(1\right)}+\ldots +n_{l}p_{succ(1)}^{\left(l\right)}=\sum_{i=1}^{l}n_{i}p_{succ(1)}^{\left(i\right)}$$

where $p_{succ(1)}^{\left(i\right)}(n_{1},n_{2},\ldots ,n_{l})$ is defined by (5).

In Figure 3, the plot of the $p_{succ}(n-m,m)$ versus *m* according to (9) for *k*_1_ = 1, *k*_2_ = 3, *n* = 5 is presented. As seen, the $p_{succ}(n-m,m)$ increases with growing *m*. Thus, the final result of moving the backlog state of any node from *k*_1_ to *k*_2_ is an increase of total bandwidth of the communication system. The corresponding conclusion is formulated in Remark 3.

**Remark** **3.** *If any node among the other contending nodes changes its backlog state, then the total probability of successful transmission in the system also changes. If a node moves toward higher backlog states, the total probability increases, and if a node moves toward lower backlog states, the total probability decreases. Thus, if n_i_ < n_j_, then*:

(10) $$p_{succ}(n_{1},\ldots ,n_{i},\ldots ,n_{j},\ldots n_{l})<p_{succ}(n_{1},\ldots ,n_{i}-1,\ldots ,n_{j}+1,\ldots n_{l})$$

Remark 3 is intuitively clear because with the growing backlog state of any node, the true contention becomes lower and vice versa. The formal proof of (10) is a relative straightforward extension of the proof of Remark 3 in [18]. In particular, by setting (9) to (10), it may be shown that:

(11) $$\frac{p_{succ}(n_{1},\ldots ,n_{i}-1,\ldots ,n_{j}+1,\ldots ,n_{l})}{p_{succ}(n_{1},\ldots ,n_{i},\ldots ,n_{j},\ldots ,n_{l})}>1$$

The cause of improving total bandwidth utilization represented by Remark 3 is that the decrease of the success probability of the node that moves toward higher backlog states (defined by Remark 2) is lower than the sum of increases of probabilities of successful transmissions gained by the other nodes that occupy their previous backlog states.

The following remark defines the upper bound of the increase of the total probability of successful transmission evoked by moving a number of nodes toward higher backlog states.

**Remark** **4.** *If the backlog state of a set of n_i_ nodes among a total number of n nodes increases significantly, then the total probability of successful transmission approaches its upper limit equal to the total probability of successful transmission for a number of n − n_i_ contending nodes*.

If the backlog state *k_l_* occupied by a set *n_l_* nodes increase significantly, then:

(12) $$\underset{k_{l}\to \infty }{\lim}p_{succ}(n_{1},n_{2},\ldots ,n_{l-1},n_{l})=p_{succ}(n_{1},n_{2},\ldots ,n_{l-1},0)$$

Remark 4 is also intuitively clear since *n_l_* nodes with growing backlog state are gradually eliminated from the contention, and the true contention is then reduced to a number of *n* = *n*_1_ + … + *n_l−_*_1_ contenders. The formal proof of (12) is a relatively straightforward extension of the proof of Remark 4 in [18].

## 5. Stability of Bandwidth Share Process

Now, we will examine the stability of bandwidth allocation process in the predictive *p*-persistent CSMA. We assume that the process of implicit bandwidth allocation is *stable* if any node that has a lower chance than a certain other contending node to transmit successfully at the same time has lower chance to additionally decrease in the near future the probability to access the channel. Similarly, the process of implicit bandwidth allocation is said to be *unstable* if any node that has a lower chance than a certain other contending node to possess the channel access, at the same time, has a higher chance to decrease in the near future the probability to access the channel successfully.

As follows from the discussion in Section 3.1, the differences between states of backlog counters may be introduced only by collisions in case if the collision detection is provided by transmitters of colliding packets (partial collision detection), or if the collision detection is absent. More specifically, if the collision detection is absent, each transmitter of a packet involved in a collision increases its backlog counter by the number (*Delta_BL*–1) where *Delta_BL* ≥ 0 is a number encoded in the header of the packet that it transmits. The packets involved in a collision may have different *Delta_BL*; thus, their senders may modify their counters by different increments. If the partial collision detection is provided, all the transmitters of packets involved in a collision increase their backlog counters by one. Both without and with partial collision detection, the nodes that do not transmit decrease their backlog counters by one. To sum up, either with partial collision detection, or with a lack of collision detection, the probability of successful transmission of the nodes involved in collisions may decrease in the near future. Conversely, the probability of successful transmission of the nodes that do not participate in collision may increase.

Thus, the probability that a particular node participates in a collision is a measure representing the potential susceptibility to decrease in the near future its probability to transmit successfully. The problem of examining the stability of bandwidth allocation is reduced to the evaluation of the collision probability against the probability of successful transmission.

### 5.1. Definition of Collision Probability

The probability $p_{coll(1)}^{\left(1\right)}(n,0)$ that a packet sent by a certain node among a number of *n* contenders is involved in a collision assuming that each node stays in the state *k*_1_, is:

(13) $$p_{coll(1)}^{\left(1\right)}(n,0)=\frac{1}{16k_{1}}\sum_{s=1}^{16k_{1}}\sum_{x=1}^{n-1}C_{x}^{n-1}{\left(\frac{1}{16k_{1}}\right)}^{x}{\left(\frac{16k_{1}-s}{16k_{1}}\right)}^{n-x-1}$$

The probability $p_{coll(1)}^{\left(1\right)}(n,0)$ is defined by summing the products of the following partial probabilities for each one from *s* = 1, …, 16*k*_1_ slots:

- probability that a distinct node selects a certain slot *s*, *s* = 1, …, 16*k*_1_, which equals 1/(16*k*_1_);
- probability that the other *x* = 1, …, *n* − 1 nodes choose the same slot *s*, *s* = 1, …, 16*k*_1_, which equals 1/(16*k*_1_)*^x^* multiplied by the binomial coefficient $C_{x}^{n-1}=(n-1)!/\left[x!(n-x-1)!\right]$ that represents the number of combinations of *x* elements from a set of (*n* − 1) elements;
- probability that the other (*n* − *x* − 1) nodes choose one from the slots *s* + 1, …, 16*k*_1_, which equals [(16*k*_1_ − *s*)/16*k*_1_]*^n^*^−*x*−1^.

Furthermore, let us consider the probability of collision in a more general scenario defined by the basic backlog inconsistency model introduced in Section 4.2 when the contending nodes occupy the backlog states *k*_1_ and *k*_2_ and the numbers of nodes in the particular state equal *n* − *m* and *m*, respectively.

If *n* − *m* = 1, the probability $p_{coll(1)}^{\left(1\right)}(1,n-1)$, that one node that is in the backlog state *k*_1_ participates in a collision if the other (*n* − 1) nodes are in the backlog state. *k*_2_ is defined similarly to (13) as:

(14) $$p_{coll(1)}^{\left(1\right)}(1,n-1)=\frac{1}{16k_{1}}\sum_{s=1}^{16k_{1}}\sum_{x=1}^{n-1}C_{x}^{n-1}{\left(\frac{1}{16k_{2}}\right)}^{x}{\left(\frac{16k_{2}-s}{16k_{2}}\right)}^{n-x-1}$$

If *n* − *m* > 1, then the probability $p_{coll(1)}^{\left(1\right)}(n-m,m)$ that a node which resides in the state *k*_1_, participates in a collision is:

(15) $$p_{coll(1)}^{\left(1\right)}(n-m,m)=p_{coll(1)}^{(1)*}+p_{coll(1)}^{(1)**}$$

where

(16) $$p_{coll(1)}^{(1)*}=\frac{1}{16k_{1}}\sum_{s=1}^{16k_{1}}\sum_{x=1}^{n-m-1}C_{x}^{n-m-1}{\left(\frac{1}{16k_{1}}\right)}^{x}{\left(\frac{16k_{1}-s}{16k_{1}}\right)}^{n-m-x-1}{\left(\frac{16k_{2}-s}{16k_{2}}\right)}^{m}$$

(17) $$p_{coll(1)}^{(1)**}=\frac{1}{16k_{1}}\sum_{s=1}^{16k_{1}}\sum_{x=0}^{n-m-1}\sum_{y=1}^{m}C_{y}^{k}C_{x}^{n-m-1}{\left(\frac{1}{16k_{1}}\right)}^{x}{\left(\frac{1}{16k_{2}}\right)}^{y}{\left(\frac{16k_{1}-s}{16k_{1}}\right)}^{n-m-x-1}{\left(\frac{16k_{2}-s}{16k_{2}}\right)}^{m-y}$$

The probability $p_{coll(1)}^{(1)*}$ defines the possibility that a given node that is in the state *k*_1_ collides with a number of *x* = 1, …, *n* − *m* − 1 nodes that are also in the state *k*_1_, while the other *n* − *m* − *x* − 1 nodes in the state *k*_1_ and *m* nodes in the state *k*_2_ lose the contention by drawing 0later slots than the slot when the collision happens. The probability $p_{coll(1)}^{(1)**}$ defines the possibility that a given node collides with a number of *y* = 1, …, *m* nodes that are in the state *k*_2_, and possibly also with *x* = 0, …, *n* − *m* − 1 nodes that are in the state *k*_1_, while *m* nodes in the state *k*_2_ and *n* − *m* − *x* − 1 nodes in the state *k*_1_ lose the contention by drawing later slots than the slot when the collision happens.

Similarly, the probability $p_{coll(1)}^{\left(2\right)}(n-m,m)$ that a node which resides in the state *k*_2_ participates in a collision is:

(18) $$p_{coll(1)}^{\left(2\right)}(n-m,m)=p_{coll(1)}^{(2)*}+p_{coll(1)}^{(2)**}$$

where

(19) $$p_{coll(1)}^{(2)*}=\frac{1}{16k_{2}}\sum_{s=1}^{16k_{1}}\sum_{x=1}^{n-m}C_{x}^{n-m}{\left(\frac{1}{16k_{1}}\right)}^{x}{\left(\frac{16k_{1}-s}{16k_{1}}\right)}^{n-m-x}{\left(\frac{16k_{2}-s}{16k_{2}}\right)}^{m-1}$$

and

(20) $$p_{coll(1)}^{(2)**}=\frac{1}{16k_{2}}\sum_{s=1}^{16k_{1}}\sum_{x=0}^{n-m}\sum_{y=1}^{m-1}C_{y}^{m-1}C_{x}^{n-m}{\left(\frac{1}{16k_{1}}\right)}^{x}{\left(\frac{1}{16k_{2}}\right)}^{y}{\left(\frac{16k_{1}-s}{16k_{1}}\right)}^{n-m-x}{\left(\frac{16k_{2}-s}{16k_{2}}\right)}^{m-y-1}$$

The probability $p_{coll(1)}^{(2)*}$ defines the possibility that a given node that is in the state *k*_2_ collides with a number of *x* = 1, …, *n* − *m* nodes that are in the state *k*_1_. The probability $p_{coll(1)}^{(2)**}$ defines the possibility that a given node collides with a number of *y* = 1, …, *m* − 1 nodes that are in the state *k*_2_, and possibly also with *x* = 0, …, *n* − *m* nodes that are in the state *k*_1_.

### 5.2. Stability of Bandwidth Share

The careful analysis shows that the maximum of the probability that a given node residing in the state *k_i_* participates in a collision is defined only by the state *k_i_* and is independent of the number of nodes and their deployment among the backlog states as is stated in the following remark. Assume that the network is characterized by the general inconsistency model introduced in Section 4.2.

**Remark** **5.** *If a certain node occupies the backlog state k_i_, then the probability that this node will participate in a collision is not greater than* 1/(16*k_i_*):

$$p_{coll(1)}^{\left(i\right)}(n_{1},\ldots ,n_{i},\ldots n_{l})\leq \frac{1}{16k_{i}}$$

**Proof.**  1. Let us consider the situation when a given node occupies the state *k*_1_ which is the lowest state among the backlog states occupied by the contending nodes. A given node participates in a collision only if it selects the same slot as the earliest slot selected by any node from a group of the other (*n* − 1) nodes:

- if at least one from the group of the other (*n* − 1) nodes is also in the state *k*_1_, the earliest slot selected by any node from this group belongs to the range of 1, …, 16*k*_1_ slots with probability equal to one. Thus, the probability that a given node selects exactly the same slot as the earliest slot selected by the station(s) from the group of (*n* − 1) nodes, which means that a collision occurs, equals 1/(16*k*_1_);
- if none of the group of the other (*n* − 1) nodes is in the state *k*_1_, then the earliest slot selected by any node from this group belongs to the range of 1, …, 16*k*_1_ slots with probability less than one. Thus, the probability that a given node that draws from the range of 1, …, 16*k*_1_ selects exactly the same slot as the earliest slot selected by the station(s) from the group of (*n* − 1) nodes, which represents a collision, is less than 1/(16*k*_1_).

Therefore, it was proven that Remark 6 is valid if a given node occupies the state *k*_1_ provided that *k*_1_ is the lowest state among the backlog states occupied by all the contending nodes.

2. Let us consider the situation when a given node occupies the state *k_i_* > *k*_1_, and there is at least one node that occupies the backlog state *k*_1_. The earliest slot selected by any node from a group of the other (*n* − 1) nodes belongs to the range of 1, …, 16*k*_1_ slots with probability equal to one. As before, a given node participates in a collision only if it selects the same slot as the earliest slot selected by any node from a group of the other (*n −* 1) nodes. This particular slot is selected by a given node with probability 1/(16*k_i_*) since it draws from a range of 1, …, 16*k_i_*. Thus, it was proven that Remark 6 is valid if a given node occupies the state *k_i_* > *k*_1_ higher than the lowest state *k*_1_ among the backlog states occupied by all the contending nodes. Thus, the proof of Remark 6 is completed. In particular, in Appendix A, it is formally proven that $p_{coll(1)}^{\left(1\right)}(n,0)=1/\left(16k_{1}\right)$, and $p_{coll(1)}^{\left(1\right)}(1,n-1)\leq 1/\left(16k_{1}\right)$. Similarly, it may be easily shown that $p_{coll(1)}^{\left(1\right)}(n-m,m)=1/\left(16k_{1}\right)$, and $p_{coll(1)}^{\left(2\right)}(n-m,m)=1/\left(16k_{2}\right)$. □

Remark 5 is conclusive for examining the stability of bandwidth allocation in the predictive *p*-persistent CSMA adopted as a random MAC protocol in LON networks. Namely, if a distinct node has lower probability to transmit successfully than the other node, then the former node must reside at higher backlog state than the latter. According to Remark 5, the node that resides at higher state has lower chance to participate in a collision, which is the only potential cause of possible decreasing in the near future its probability to access the channel successfully as discussed above in Section 4. Hence, the process of implicit bandwidth allocation in the predictive *p*-persistent CSMA scheme is stable.

## 6. Conclusions

The paper presents an analytic approach to qualitative evaluation of the media access unfairness of random media access in Local Operating Networks (LON), which is one of commercial platforms of Industrial Internet of Things (IIoT). The unfairness in accessing the LON channel is introduced by a collision avoidance mechanism in the predictive *p*-persistent CSMA protocol adopted at the media access control level. It has been shown that the channel access in LON systems is unfair in the short term for medium traffic load conditions, and in the long term for heavy loaded network. Furthermore, it is explained that the average bandwidth allocated to a particular node is determined implicitly by the traffic load scenario (i.e., rate and specification of input traffic generated to the network by particular nodes in the network). Finally, it is demonstrated that possibly unfair bandwidth share in LON networks determined implicitly by load scenario is stable because with lowering a fraction of actual network bandwidth accessible by a given station, the probability to decrease it in the future also drops. The presented analysis exemplifies the tradeoff between fairness and system efficiency, which is a common paradigm in communication systems. The work contributes to rapidly growing research on fairness of various network services including Internet-of-Things-based networks.

## Appendix A

The proofs that:

(A1) $$1.\;p_{coll(1)}^{\left(1\right)}(n,0)=\frac{1}{16k_{1}}$$

**Proof.** On the basis of (13):

(A2)
$$p_{coll(1)}^{\left(1\right)}(n,0)=\frac{1}{16k_{1}}\sum_{s=1}^{16k_{1}}\sum_{x=1}^{n-1}C_{x}^{n-1}{\left(\frac{1}{16k_{1}}\right)}^{x}{\left(\frac{16k_{1}-s}{16k_{1}}\right)}^{n-x-1}$$

On the basis of Newton’s generalized binomial theorem:

(A3) $$\sum_{x=0}^{n-1}C_{x}^{n-1}{\left(16k_{1}-s\right)}^{n-x-1}={\left(16k_{1}-s+1\right)}^{n-1}$$

Conversely:

(A4) $$\sum_{x=1}^{n-1}C_{x}^{n-1}{\left(16k_{1}-s\right)}^{n-x-1}=\sum_{x=0}^{n-1}C_{x}^{n-1}{\left(16k_{1}-s\right)}^{n-x-1}-{\left(16k_{1}-s\right)}^{n-1}$$

Thus:

(A5) $$\sum_{x=1}^{n-1}C_{x}^{n-1}{\left(16k_{1}-s\right)}^{n-x-1}={\left(16k_{1}-s+1\right)}^{n-1}-{\left(16k_{1}-s\right)}^{n-1}$$

and next

(A6) $$\sum_{s=1}^{16k_{1}}{\left(16k_{1}-s+1\right)}^{n-1}-{\left(16k_{1}-s\right)}^{n-1}={\left(16k_{1}\right)}^{n-1}$$

Finally:

(A7) $$\sum_{s=1}^{16k_{1}}\sum_{x=1}^{n-1}C_{x}^{n-1}{\left(\frac{1}{16k_{1}}\right)}^{x}{\left(\frac{16k_{1}-s}{16k_{1}}\right)}^{n-x-1}=1$$

Thus, the proof is completed.

(A8) $$2.\;p_{coll(1)}^{\left(1\right)}(1,n-1)<\frac{1}{16k_{1}}$$

□

**Proof.** On the basis of (13):

(A9)
$$p_{coll(1)}^{\left(1\right)}(1,n-1)=\frac{1}{16k_{1}}\sum_{s=1}^{16k_{1}}\sum_{x=1}^{n-1}C_{x}^{n-1}{\left(\frac{1}{16k_{2}}\right)}^{x}{\left(\frac{16k_{2}-s}{16k_{2}}\right)}^{n-x-1}$$

Since by the assumption of *k*_2_ > *k*_1_, thus:

(A10) $$\sum_{s=1}^{16k_{1}}\sum_{x=1}^{n-1}C_{x}^{n-1}{\left(\frac{1}{16k_{2}}\right)}^{x}{\left(\frac{16k_{2}-s}{16k_{2}}\right)}^{n-x-1}<\sum_{s=1}^{16k_{1}}\sum_{x=1}^{n-1}C_{x}^{n-1}{\left(\frac{1}{16k_{1}}\right)}^{x}{\left(\frac{16k_{1}-s}{16k_{1}}\right)}^{n-x-1}$$

Finally:

(A11) $$p_{coll(1)}^{\left(1\right)}(1,n-1)<p_{coll(1)}^{\left(1\right)}(n,0)$$

Thus, the proof is completed because of (A1). □
